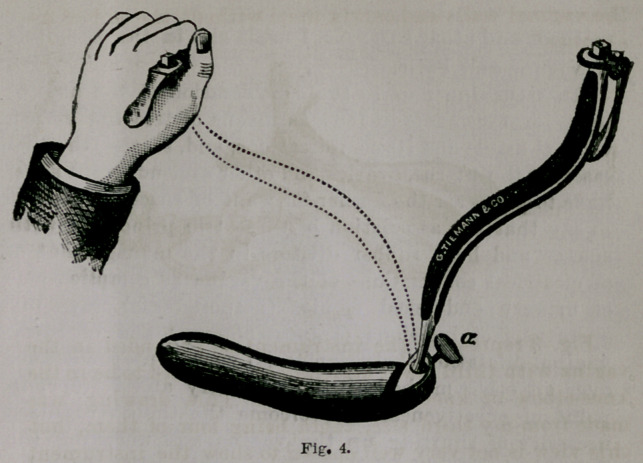# The Value of Graduated Pressure in the Treatment of the Vagina, Uterus, Ovaries and Other Appendages

**Published:** 1882-12

**Authors:** Nathan Bozeman

**Affiliations:** New York, Surgeon to the Woman’s Hospital of the State of New York


ATLANTA
MEDICAL REGISTER.

VOL. II.] DECEMBER, 1882. [No. 3

### Original.

### THE VALUE OF GRADUATED PRESSURE IN THE TREATMENT OF DISEASES OF THE VAGINA, UTERUS, OVARIES AND OTHER APPENDAGES.

BY NATHAN BOZEMAN, M.D., NEW YORK,
Surgeon to the Woman's Hospital of the State of New York.

In THE ATLANTA MEDICAL REGISTER for September, 1882, there appeared an article entitled "The Application of Pressure in Diseases of the Uterus, Ovaries and Peri-Uterine Structures, by V. H. Taliaferro, M.D., Atlanta, Ga., Professor of Obstetrics and Diseases of Women and Children in the Atlanta Medical College," in which I am taken to task by the learned Professor in not over-choice language because I did not credit him with the merit he thought he deserved, in a paper which I submitted to the American Gynæcological Society at its annual meeting in Philadelphia, September, 1878.

I am sorry the Professor deemed it necessary to resort to so questionable a mode of enforcing his views or practice upon the notice of the profession, since it deprives me of the pleasure of treating his complaints with that high degree of consideration which I always try to accord to my colleagues who may chance to differ from me on topics of common interest.

In order to make myself fully understood, and to give some idea of the successive steps by which I was led, in 1878, to set forth my views in the paper referred to, entitled "The Mechanism of Retroversion and Prolapsus of the Uterus Considered in Relation to the Simple Lacerations of the Cervix Uteri and their Treatment by Bloody Operations," it will be necessary for me to speak somewhat in detail of my inventions and improvements in instruments, and the various modes of using them, before I enter upon my subject.

As far back as 1855 I had learned from experience that the cicatricial bands of the vagina, found as complications of vesico-vaginal fistule, could be divided with the knife and the expansion of the organ, through graduated pressure with bits of sponge packed in oil-silk bags as dilators, could be carried to a degree often far beyond the normal caliber of the organ with corresponding loosening and elevation of the uterus and its appendages when fixed as results of pelvic inflammation. These sponge dilators were of two kinds: first, vulvo-vaginal; and second, intra-vaginal, the length and size being determined always by the depth of the vagina, and degree of contraction and resistance to be overcome.

This principle of dilatation of the vagina by graduated sponge pressure, associated with my button suture principle and the old knee-elbow position, marks the era (May, 1855,) of the first consecutive successes in a series of seven operations for vesico-vaginal fistule to be found upon record. As a point of historical interest I will here quote from the first published report of the successful employment of my sponge dilators as a means of making direct pressure upon the walls of the vagina and upon the uterus, in a case of two vesico-vaginal fistules and almost complete obliteration of the vagina just below the cervix uteri: "A fistulous opening, three-quarters of an inch in length, occupied the vesico-vaginal septum and extended from near the beginning of the urethra obliquely upwards and to the left, terminating abruptly at the point of coarctation. Here a careful examination revealed a small opening, which allowed a probe to pass into the vaginal

*cul de sac* above, and from thence into the bladder, showing clearly that another fistule existed in this situation. Having thus ascertained the true condition of things, I became satisfied that two operations would be required.

"In a few weeks (after my first fistulous closure, June 12th, 1855,) I made preparation for the other (the second) operation by first *breaking up the morbid adhesions between the two walls of the vagina, so as to expose the fistulous opening above. To prevent reunion of the parts, a bag made of oil-silk and stuffed with bits of sponge was introduced into the vagina.* This was removed daily, and injections of cold water used, by which means the upper extremity of the vagina was, in a few weeks, dilated to its normal size, and the fistule exposed."

August 23d, this second fistule was closed at a single operation with my button suture, and the cure thus completed.

(See Case II, *Louisville Review*, May, 1856.)

Next I associated with these two new principles of practice a third, the drawing down of the uterus when fixed and immovable (hysterœphelcosis) after incisions and dilatation (kolpostenotomy and kolpoecpetasis), in order to make it subservient to the closure of large fistules, and thus was inaugurated a method which superseded the necessity of the dangerous plastic procedure of Jobert de Lamballe—*autoplastie par glissement.* Fourthly, with these three principles combined, I next undertook to overcome retroflexion of the uterus, with fixation and displaced ovaries superinduced by incarceration of the cervix uteri in the bladder, as a preparatory measure. The result was a complete restoration of the uterus and its appendages to their proper positions, and the final closure of the associated fistulous orifice.

(See Case XXXVIII., *New Orleans Medical and Surgical Journal*, May, 1860.)

By this latter combination of principles I cured five cases out of six; no other operator, as far as I am aware, having ever recorded, even at the present time, a case on this basis of maintaining the normal outlet of the catamenia.

In 1858, or about this time, Dr. Sims modified my principle of intra-vaginal dilatation through graduated sponge pressure in oil-silk bags, by using a *glass plug* instead, intended simply for vulvo-vaginal dilatation, but this was far inferior to my sponge dilator, on account of its more limited application in the graver complications of vesico-vaginal fistule.

In 1867, in a class of inaccessible vesico-vaginal fistules occurring in vaginas of very large size and quite relaxed, I discovered the total worthlessness of even the larges size of my modification of the univalve speculum, even the same size of Dr. Sims' modification of it, for operative purposes in the old knee-elbow position. And in order to meet the emergencies then presented, I devised a new self-retaining spring speculum for dilating such vaginas to any desired extent, and at the same time a supporting and confining apparatus for securing the patient in the fixed *knee-chest position*, that gave me absolute control over patient and fistule without the aid of assistants. These two additional inventions and the anatomical considerations suggesting their adaptation to use, will be better appreciated by reading the following extract from a publication of mine upon these points, entitled "A Spring and Self-Retaining Speculum," to be found in the *New York Medical Record*, January 1st, 1868:

"The vagina, as a membranous canal, in the distended state may properly be said to represent a truncated cone with the base turned upward and the apex downward, corresponding with its mouth.

"The general outline of the organ, as viewed in its natural condition, is such as would result from bringing the two opposing walls of the cone together, the cervix uteri being encircled by it at the center of its base, and its mouth closed by the falling together of the labia majora.

"The line, therefore, formed by the anterior and posterior walls of the organ coming together is transverse, while that formed by the opposing surfaces of the labia is antero-posterior, being at right angles.

"Now the most natural indications for the dilatation of this canal with the peculiarities named, would appear to be, first, separation of the labia, and second the two opposing walls of the collapsed cone, so to speak. This, scarcely need I say, is the view generally taken of the relationship of these parts, and the usual practice is based upon it of bringing within the field of observation the cervix uteri and the two vaginal walls.

"This plan of antero-posterior dilatation of the vagina, it matters not what form of speculum is used, I conceive to be a popular error, and it is wholly at variance with the true anatomical relationship of the parts. I shall presently attempt to explain more fully my meaning in my description of a new form of speculum, which I have the pleasure of presenting now to the notice of the profession. The principle of construction, as well as principle of action of this new instrument, will be found to differ from all others heretofore in use in several respects, which I shall explain farther on. Suffice it to say, one of the essential differences is in what might be termed the working point of the instrument, that portion which is applied to the resistance. The blades of my instrument are introduced between the opposing walls of the vagina edgewise instead of flat, as formerly; and the dilatation is effected transversely or horizontally, as will be better understood when I come to explain the principle of action. The same instrument applies to the dilatation of the vulva as the vaginal canal; thus giving at one glance a view of the parts from the mons veneris to the cervix uteri in front; and behind, of nearly the whole posterior wall of the vagina—every point within this extensive range being made accessible for operative purposes.

"The dilatation thus effected is so regulated that the labia and the two extremities of the vagina are put upon the stretch only to the extent desired, which is in strict accordance with the anatomical conformation of the parts, this being of such a nature as to make the instrument *self-sustaining*, one of its peculiarties; another being *elasticity of flexure.* This principle of elasticity has never before been embodied in any form of speculum as far as I am aware, and its utility and importance in my judgment, cannot be too highly estimated. Instead of the hard inflexible blades (of the bivalve, trivalve and quadrivalve instruments) formerly used, touching only at one or two points the soft and delicate structures, we have now the soft, elastic spring adapting itself to all the points of resistance with a uniformity to be attained in no other way.

"The indications for complete dilatation of the vagina and vulva, I conceive to be four:

1. Elevation of the perineum.

2. Elevation and support of the upper part of the posterior wall of the vagina.

3. Transverse dilatation of the labia majora and the mouth of the vagina.

4. Distention and steadiness of the upper part of the anterior wall of the vagina, the vesico-vaginal septum.

"These are the indications to be fulfilled, according to my judgment, independent of any and all efforts of the patient to the contrary; and any instrument, whether *self-retaining* or *not*, that does not meet these ends must be regarded as incomplete. With my instrument I claim the accomplishment of all, the *fulfillment of the third and fourth indications* being an advance beyond other methods, to say nothing of the self-retaining quality of the instrument which is based upon more correct principles than any plan heretofore presented to the notice of the profession.

"As regards the position, support and confinement of the patient, I propose a few remarks before entering upon the description of my instrument, as I consider these points of no little consequence in certain operations, especially those upon the anterior wall of the vagina.

"While my speculum is equally well-adapted to all positions, I prefer in the description and application of it, to consider the patient resting upon her knees and breast, the body forming a right angle with the thighs, and the thighs a right angle with the legs. This position I now prefer to all others and with propriety it may be termed *the right-angle position upon the knees.*

"In no other position, according to my judgment, whether chloroform be used or not, can the patient be made so comfortable and secure without the aid of assistants. My supporting apparatus for this position, when folded up, is compact, light and portable, weighing only eleven pounds. It exceeds twelve inches in height only on one side, the depth and width being twelve by eighteen inches. I hope before long to publish a description of this *thoracic rest or support.*"

In the *New York Medical Journal* for February 1859, in an article entitled: "Remarks on the Advantages of a Supporting and Confining Apparatus, and a Self-retaining Speculum in the Operation of Vesico-Vaginal Fistule; Modes of Certain Forms of Suture; Their Results Practically Contrasted in the Same Cases and upon the Same Fistulous Openings," I introduced a cut to show the knee-

Fig. 1.

chest position of the patient upon my Supporting and Confining Apparatus and the principal objects sought to be attained by it, to wit:

1. "Extension of the vertebral column and relaxation of the abdominal muscles essential to free gravitation forward of the pelvic and abdominal viscera.

2. "Support and mechanical confinement of the patient by controlling muscular action at certain points without encumbering the abdomen, or interfering with the functions of respiration and circulation.

3 "The safe administration of anæsthetics."

Fig 1, shows the apparatus at work in the knee chest position.

Fig. 2.

Fig. 2, shows the exaggerated knee-elbow position.

In regard to the latter position I would say as viewed from an historical standpoint, it came into use cotemporaneously with that of the knee-elbow position, since it is well known by all who have had any experience with the knee-elbow position that a patient when placed in it for examination or operation almost always sinks from fatigue and exhaustion into the exaggerated knee elbow position as above shown and therefore the advantages and disadvantages of it must have been long and well understood in practice.

From the beginning of my experience with the knee-elbow position (1853) my object always was to prevent the patient from getting into this exaggerated knee-elbow position, which I effected by placing a support under the chest so as to bring the body up to a horizontal plane as shown in Fig 1. In this way I avoided one of the disadvantages of the position, perhaps the most important, namely, the cutting off of the light from the vesico-vaginal septum and cervix uteri. My supporting and confining apparatus, as here illustrated, was simply an improvement upon my simple bench support usually extemporized for the occasion of converting an exaggerated into a knee-chest position.

Dr. Henry F. Campbell, from whose article I have copied this cut, which was published in the *Transactions of the American Gynæcological Society* for 1876, seven years later, and entitled "Pneumatic Self-replacement in Dislocations of the Gravid and Non-gravid Uterus," must also have known the fact here stated, and yet he claimed it as something which had scarcely been known, up to the time of his writing, for practical use in the treatment of prolapsus and retroversion of the uterus. Not only this, he named it the Genu-pectoral Position, the English designation of my position, the knee-chest, published nine years before; and what is still stranger, without making any acknowledgement or explanation for so doing. In all of my references, therefore, to the *knee-chest* position in these remarks, I mean the one with the body of the patient resting on a horizontal plane upon my supporting and confining apparatus or any improvised support, and the *exaggerated knee-elbow position* with the breast of the patient on the same plane with the knee, as here illustrated by Dr. Campbell.

I also described in the same number of this journal, in connection with my knee-chest position, further improvements and the completion of my self-retaining speculum setting forth again its "principal peculiarities" in these words:

1. "The system of leverage employed, which gives us increased power over increased resistance.

2. "Transverse dilatation with uniformly varying movement of the blades, which gives us a thin and favorable form of its points for introduction, and a reversal of the size of the two extremities of the instrument when expanded within the vagina. By virtue of this *flaring expansion of the blades within the ascending rami of the ischia*, the instrument is made *self-retaining*, which distinguishes it from all others of this class previously constructed.

3. "The *elasticity of flexure* belonging to the working-part of the instrument, which gives it an easy adaptation to the soft structures, both of the vagina and vulva. This is also a feature of the instrument that particularly distinguishes it from other valved specula, heretofore in use.

4. "The applicability of it in all positions, and the advantages secured to the physician or surgeon, of making all examinations, or of doing all operations required upon the vaginal walls and cervix uteri without the aid of assistants."

Fig. 3.

Fig. 3 represents the instrument as expanded in the vagina with third blade attachment, supposed to be in the knee-elbow or knee-chest position. This drawing was made from my third size, there being four of them, but this view is not very well chosen to show the instrument to advantage. The second size speculum is the one suitable for the ordinary treatment of uterine diseases in the recumbent, knee-elbow and knee-chest positions without an assistant.

Although my speculum at this stage of its completion answered all purposes for which it was intended and even more, as I shall presently show, I found the scope of its usefulness, especially in the knee-elbow and knee chest positions could be greatly increased by the addition of an independent perineo-rectal elevator in the place of the third blade attachment, now found better suited as a rectal depressor in the recumbent posture. With this perineal elevator or retractor set upon a curved handle at different angles with sufficient size to allow the proper grasp of the surgeon's hand, it was possible not only to raise the perineum and the already expanded speculum to the highest point and to throw the greatest amount of light upon the anterior wall of the vagina and cervix uteri, but to expose at the same time for operative purposes the posterior wall as well. The blade was narrow, thin, almost flat and slightly curved on its convex side, independent of the blades of the speculum and free in its backward and forward movements between the latter for introduction or removal.

Fig. 4.

Fig. 4 shows the instrument at two set angles. For the posterior wall of the vagina, as a depressor, it admits of two other set angles making in all, four.

Accompanying the speculum and perineal elevator, for use in all positions without an assistant, there are : *a.* a narrow smooth spatula with straight handle ; *b.* a narrow double-hook spatula also with straight handle, and *c.* a pair of curved uterine forceps. These five instruments constitute the set as now in general use.

The instrument set at all four angles with my speculum and supporting apparatus is figured in the second edition of Dr. Frank H. Hamilton's work on the "Principles and Practice of Surgery," 1873. On page 919 he shows the supporting and confining apparatus with patient in position as "Bozeman's Knee-Chest Position."

It was with these instruments above described, and my knee-chest position that I met the late Prof. Gustav Simon of Heidelberg, in the autumn of 1874, and entered with him, in the hospital of the University, a competitive trial as to the relative advantages of our respective procedures for the cure of vesico-vaginal fistule and its complications. With what result, is to be seen in the medical literature of Europe. The same trials of my procedure with other methods and other surgeons, I made also in the hospitals of Vienna and Paris.

In returning now to the consideration of the uses of these improvements in 1869, as means of treating vesico-vaginal fistule and its complications, and, second, the diseases of the uterus, ovaries and other appendages, I shall have to pass over the former for want of space. Suffice it to say that the association of these two principles with sponge and hard rubber dilators in the management of of cicatrical contractions of the vagina as complications of urinary and fæcal fistules (kolpostenosis) gave my operations of incisions (kolpostenotomy) and of intra-vaginal dilatation (kolpoecpetasis) such a preponderance over all other methods in this country and Europe, in point of effectiveness to overcome the then recognized necessity of shutting up the vagina and unsexing the individual (kolpokleisis), as might well be called a revolution in vaginal surgery.

The proof of this is to be found in the statistics of the late Prof. Simon, of Heidelberg, the originator of kolpokleisis, who subjected 34 per cent. of his cases at that time to this method of treatment, and that now, only eig t years after I introduced my method into the hospitals of Heidelberg, Vienna and Paris, there is scarcely to be found, in Austria and Germany, a surgeon who openly advocates kolpokleisis. Of the physicians of New York who frequently witnessed my operations about this date (1869), I will mention Drs. Frank H. Hamilton, I. E. Taylor, S. T. Hubbard, G. Sabine, T. C. Finnell, J. F. Chauveau and M. J. Moses.

*My Speculum and the Knee-Elbow and Knee-Chest Positions as Means of Treating the Diseases of the Uterus, Ovaries and Other Appendages.*—Scarcely need I say that ten or twelve years' experience in the treatment, especially of retroflexion of the uterus with fixation as a complication of vesico-utero-vaginal fistule, previous to 1869, was quite enough to convince me of the superiority of my speculum over the univalve in all positions of the patient for treatment of the diseases in question, and as for the usual working positions—*the decubitus and knee-elbow, without an*

*assistant*—my speculum, I felt, admitted of no comparison with the univalve. In cases of this class I had found it always necessary not only to maintain a full lateral dilatation of the vagina through sponge pressure, but to gradually increase the latter in the linear or perpendicular direction according to the commencing movement and elevation of the uterus from its fixed position against the rectum. This insured gradual stretching or elongation of the posterior wall of the vagina, and favored direct pressure upon the uterus and other resisting points in and about the broad ligaments and ovaries. In this way I found it to be possible, after making the necessary incisions for the disengagement of the cervix uteri from the bladder, to stretch the posterior wall of the vagina in extreme cases, elongating it from two and a half to five and a half inches.

It was not, therefore, difficult to modify this form of graduated lateral and linear pressure to suit retroflexions of the uterus with fixation, unattended by lesions of the vesico-vaginal septum.

The modification simply consisted in making the oil-silk bags narrower and not stuffing them so firmly, the object being here to lessen lateral and increase linear pressure. Made in this way, the cylinders admitted of easier introduction and removal, and could, when it was desirable, be flattened, so as to give them a form even more conducive to the avoidance of pressure upon the bladder and rectum. The *point d'appui* within the pubic arch and perineum, of course, remained the same. The rule, likewise, of using the warm douche twice a day, and of cleansing the sponges once a day, or sometimes every other day, was strictly observed. When it was not possible to elevate the uterus to its normal position by this procedure, supplemented by the use of the uterine sound, as very often happened in my early experience, and a Hodge's pessary could not be worn, I would continue the cylinder in its stead, which, after the active treatment, could be easily managed by the patient herself. In the active treatment I found my thin-bladed perineal elevator used alone to be of the greatest service, since it greatly facilitated the introduction of the cylinders in the knee-elbow position, because I found it more convenient and suitable than my knee-chest or the exaggerated knee-elbow position, which was always more disagreeable and uncomfortable to the patient. I would very often use my knee-chest position, however, when it was an object to save the patient from the disagreeableness of putting her head below the plane of the body. For the ordinary examinations with my speculum, and even with the perineal elevator alone, this position was all that was required. For its ready utilization, all I had to do was to improvise a chest support, which I did by simply placing a few books with the pillow or cushion at hand on a couch, table or office chair, and requiring the patient to place her elbows on them, thus bringing her body up to the horizontal plane.

Of course the great object in employing any one of these positions was to enable me to judge accurately of the space to be occupied by the flattened sponge cylinder or compressor and to apportion it accurately to the degree of hard pressure that could be borne by the patient.

After the dislodgement of the uterus, by this mode of applying graduated pressure, from its fixed position against the rectum, and the replacement of the organ by the uterine sound, where this was practicable, I would adjust in the usual way a Hodge's pessary and complete the cure.

When the latter instrument, however, could not be borne, which was frequently the case, I would continue moderate pressure by the flattened sponge cylinder as a pessary in accordance with the rules before mentioned. Not only did I employ these sponge cylinders as soft pressure pessaries for retroflexion of the uterus with fixation, but also for retroversion when a Hodge's hard pressure pessary could not be tolerated owing to sub-involution and tenderness of the uterus, displacement of the ovaries or other complication. In this way great relief was afforded the sufferer, and she was saved the necessity of the constant care of her physician, as she could make and introduce the cylinders herself.

It was, therefore, by persevering efforts thus directed that I was enabled to secure results of beneficial utility and importance, which were impossible before by the use of Hodge's pessary alone. Besides, a closer study now, of the pathology of retroflexion of the uterus with fixation satisfied me that the immobility of the organ did not always result, as was then generally believed, from adhesion between the fundus of the uterus and the anterior wall of the rectum, or rather the opposed surfaces of Douglas' fossa, but from thickening and shortening of one or both of the broad ligaments, arising from pelvic cellulitis and peritonitis which were amenable to successful treatment in a very large proportion of cases by this mode of graduated and elastic linear or perpendicular pressure as above advocated.

In 1869, when I began to use hard rubber dilators, in place of the sponges in oil-silk bags in order to avoid the trouble of the latter, I tried to utilize them also in the treatment of retroflexion with fixation; but the result was not satisfactory, owing to their want of elasticity and the discomfort they generally caused the patient by undue pressure upon the rectum, bladder and urethra when the diameter or length happened to be a little too great for the space they were intended to occupy in the vagina. It is true they had the advantage, in common with the Hodge's pessary, of being less troublesome to both physician and patient, but they were found to be far inferior for the reasons stated, and the fact of their being slower in the accomplishment of the same end, the softening and stretching of the tissues around the uterus and appendages. From these considerations, I gradually laid them aside as means by which retroflexions of the uterus could be satisfactorily treated. As a valuable means, however, of treating the complications of vesico-vaginal fistule they were continued just the same.

In the hope, therefore, of finding some other material better suited, than hard rubber or metals, to supplement or take the place of my sponge cylinders in oil-silk bags with which to make graduated pressure, I turned my attention to dry cotton on account of its softness and elasticity, although in the latter quality differing widely from that possessed by sponge.

I had, in my earlier experience in the treatment of prolapsus and ulcerations of the os uteri with the old cylindrical speculum in the recumbent position, been accustomed to introduce into the vagina, after my applications of nitrate of silver, columns of dry cotton. This I did by taking pieces or balls the size of a pullet's egg, with loops of thread thrown around them with ends five or six inches long for removal, and with long straight forceps crowding piece after piece into the speculum until it was filled for two and a half or three inches. This being completed, and the end of the column steadied by the forceps held in the right hand, the speculum would be slowly withdrawn with the other hand, thus emptying the instrument of its contents and leaving in the body of the vagina the column thus constructed, with all the ends of the looped threads hanging out of the vulva to enable the patient to remove it herself. Thus was placed a cylindrical column of dry cotton, intended to elevate and keep up the uterus and its appendages, for a time, at least, with the pubic arch and perineum acting as the point of support.

This column of cotton, in the milder forms of prolapsus uteri, afforded very considerable support, and, I found, often gave great relief to the sufferer. The patient was required to remove it at the end of thirty-six or forty-eight hours, and use the warm vaginal douche. At the end of three or four days, another application of caustic would be made and the column renewed as before described.

This column, from being dry, applied itself closely to the walls of the vagina, and, from its being slow to absorb the secretions, it maintained for some time its linear resistance to the superincumbent weight of the uterus. Being, however, essentially cylindrical, and too small to fill the upper part of the vagina as applied in decubitus, it did not give the full amount of support required to the uterus and its appendages, and consequently was defective and limited in the scope of its usefulness.

Seeing, therefore, the superior advantages dry cotton offered over the old methods in the treatment of the ordinary diseases of the uterus and its appendages with the cylindrical speculum, I naturally used it after the invention of my self-retaining speculum. I also tried wool, knowing that it possessed equal elasticity, or even more than dry cotton, but patients so often complained of its harshness and its irritation of the vagina, that I gradually gave up its use, employing only the cotton, which I found to be free from these objections.

With my self-retaining speculum and perineal elevator, with the patient in the knee-elbow position, I soon discovered that I could introduce between the two walls of the vagina a flattened column of cotton, in the manner of padding. The pubic arch and perineum I considered the points of natural support. The inverted cone shape of the vagina, to which my speculum had been adapted by the flaring expansion of its blades, particularly favored this mode of using the flattened or pad form columns of dry cotton, and I soon found that my patients could not only bear this form of support better than the soft sponge cylinders, but could walk and exercise with far less inconvenience and fatigue. Besides this, I also found that this plan was attended with a great deal less trouble to the patient and myself. All the patient now had to do after the stipulated time was to remove the deranged or broken-down column by drawing the threads looped around the several pieces of cotton used, and then to take, as usual, the warm vaginal douche. This I required to be done at the end of thirty-six or forty-eight hours, according to the necessity arising from leucorrhœa or other causes. I repeated the columning, at first, very much, as I had formerly been accustomed to do when using the cylindrical columns of cotton through the old glass speculum, about every three or four days, but afterwards reduced the time, as stated.

It will suffice here to say that it was not long, after beginning this new mode of treatment, before I learned that a larger proportion of retroflexures of the uterus, whether simple or complicated, could be managed even more satisfactorily by dry cotton pressure than by sponge pressure, because it admitted of a more extended application, and could be so modified in each case as to meet the peculiarities presented without causing discomfort or unneccessary suffering to the patient. From these circumstances I gradually came to employ it in all forms of uterine, para and peri-uterine disease connected with displacements and distortions, very much as I had been accustomed to use sponge dilators in oil-silk bags for the complications of vesico-vaginal fistule; but, owing to my lack of hospital facilities, it was several years before I came to realize fully the great value of the principle of dry cotton columning associated with my self-retaining speculum and the knee-elbow or knee-chest position.

I will here describe the *modus operandi* without an assistant. The patient being in the knee-elbow or knee-chest position, and the vagina dilated laterally to the required extent with my second sized speculum and backward and forward moving perineo-rectal elevator, the latter held in my left hand, while standing at the left side of the patient, I seize, with my curved uterine forceps held in the right hand, the first piece or ball of dry cotton looped with thread, and place it at the top of the posterior *cul de sac*, or against the posterior surface of the retroflexed uterus, as the case may be. A second, third and fourth ball follow in rapid succession, and are placed and compressed with the forceps so as to occupy the entire width of the space behind the stationary points of the lateral blades of the speculum, when the whole is caught on the end of the backward and forward moving perineo-rectal elevator and held in position until several other balls are consecutively compressed and caught, thus avoiding, in every movement of the forceps and perineal elevator, direct force backwards or forwards against the rectum or bladder. The flattened column or pad of cotton, thus begun, with its broad base and now brought downwards and forwards, slightly narrowed to come within the body of the flaring blades of the speculum, is next extended onwards obliquely across the axis of the speculum, until the *point d'appui* is reached just within the pubic arch, and the contracted range of the perineum, at about the junction of the upper two-thirds of the vagina with the lower third. In course of the construction, piece by piece of the column, the flaring blades of the speculum fulfill most important ends: *a.* in securing and maintaining uninterruptedly complete separation of the lateral walls of the vagina; *b.* in distending and steadying absolutely the vesico-vaginal and recto-vaginal septa by joint action with the perineal elevator; *c.* in presenting for working purposes, by virtue of their flaring expansion, an inverted cone in accordance with the anatomical requirements of the parts; *d.* in serving as a guide and as a protection against undue lateral pressure, and consequent unpleasantness and pain to the patient; *e.* in guarding against undue pressure by the column upon the bladder or rectum, or both, by virtue of lateral distension of the anterior and posterior walls of the vagina instead of antero-posteriorly, as with the perineal elevator alone or with the univalve speculum; *f.* in allowing the firmest transverse compression of the column in its lower two-thirds, thus leaving the upper third soft and free for upward distension, pressure and movement of the uterus and ovaries, whether simply impacted or fixed; *g.* in adapting to the two laterally distended walls of the vagina a soft, dry, elastic and absorbing column of flat or pad form, wide at its upper extremity, where pressure is most needed, and narrow at its lower end, where the urethra opposes it; *h.* and in permitting itself to be removed, without derangement of the column, by simply steadying the lower end of the column with the uterine forceps still held in the right hand and inclining it backwards, and collapsing the blades with the other hand while reversing the screw.

It is proper to state that whatever medication to the cervix uteri is thought to be advisable, it is applied upon or in a special pad of cotton through the agency, usually, of glycerine and then placed in position as preparatory to the step of columning. The column is allowed to remain in position for thirty-six hours usually, when it is removed by drawing the threads left for that purpose. When by rest of the patient, coupled with the warm vaginal douches, for another thirty-six hours, the column is renewed as before, and so on to the completion of the cure.

As an illustration of my early appreciation of the principle above applied in practice, I will cite here a somewhat unique case of retroflexion of the uterus with fixation and supposed prolapsus of the ovaries, which came under my observation March 13th, 1874.

Mrs. S., aged 32, married eight or ten years, no children, stout and seemingly in robust health, consulted me on account of her extreme nervousness and inability to ride in a carriage or omnibus for nearly a year. Any quick movement of the body, as in sitting down, was intensely disagreeable, but the distress caused by riding over rough streets in an omnibus was agonizing. Sometimes, so sudden and intense would be the pain, she would involuntarily spring to her feet and abruptly leave the vehicle, in the greatest embarrassment. The cause seemed to her, she remarked, a "soreness of the womb." She had been under the treatment of several eminent physicians of New York without deriving any benefit, and having suffered so much from the trial of hard pessaries, one after another, in addition to her other troubles, she was quite disheartened as to the prospect of being cured.

An examination in the knee-elbow position, by digital and speculum exploration, readily revealed what I had suspected from the symptoms, namely: retroflexion of the uterus with fixation. There was prolapsus of one ovary; this being also slightly fixed behind the uterus. My columning of the vagina was at once begun, and then kept up, as above described, for nearly two months, with the result of restoring the uterus and ovaries to a fair position and relieving entirely all her pains and nervous symptoms. Being prevented from following up the case on account of my visit to Europe, I ordered an intra-vaginal sponge cylinder in oil silk (for day use only) to be worn for two or three months as a Hodge's pessary. It acted splendidly from the first introduction.

In August, 1877, something over three years after the treatment was discontinued, this lady called to see me, having just returned from San Francisco, and she said her health had remained perfect from the time of her discharge. An examination now showed that the axis of the uterus was above a horizontal line and that the ovaries, so far as could be determined by the finger, were out of harm's way. She remarked that she had been able, ever since my treatment, to ride in a carriage or omnibus as far and as long as any one without discomfort.

Dr. F. W. Owen, of Morristown, N. J., placed another case under my care about the same time I began the treatment in the above case. Here, there was left latero-retroflexion of the uterus with fixation, and with imprisonment of the corresponding ovary. Columning of the vagina by my method was recommended and carried on jointly by Dr. O. and myself for several months, with the result of relieving almost entirely the confined position of the uterus and restoring the patient, after a year or two, to almost robust health. In connection with the columning with dry cotton there was used also, from time to time, my intra-vaginal sponge cylinder. The latter was finally used alone in place of the Hodge's pessary which could not be borne.

Dr. J. F. Chanorin, of this city, also consulted me about the same time (March, 1874) with regard to a case of retroflexion of the uterus with slight fixation, occurring as a result of a miscarriage at sea which was followed by a mild attack of pelvic cellulitis. This took place three or four years previously, and the lady not having become pregnant since believed that she labored under some serious uterine disease, which induced her to apply for medical treatment. I recommended to the Doctor columning of the vagina with dry cotton according to my method, which he understood very well from having frequently witnessed its application in my practice. The treatment was continued about two months, at the end of which time the uterus was found quite restored to its normal position. No pessary or sponge cylinder was used as after-treatment.

Pregnancy soon followed and in due course of time a fine healthy child was born without difficulty. The patient, I learned from the Doctor a few days ago, remains after eight years perfectly well, and has given birth to another child.

Owing to the loss of health now (July, 1874) I visited

Europe and during the three years I was abroad, there was almost a complete suspension of my labors as regarded the employment of my new method of treatment for uterine and ovarian displacements. I saw but one case during the time I was away from New York and that was in the person of a young German lady, laboring under retroflexion of the uterus with fixation, whom I saw in consultation, October, 1874, with Dr. Muller of Coburg, Germany. I recommended my method of treatment, but it was not properly carried out and consequently no decided benefit resulted from it.

In June, 1877, just after my return from Europe, I was appointed consulting surgeon to St. Elizabeth's Hospital of New York, under the direction of Dr. O. Sprague Paine, Surgeon in Chief. The Doctor then had a very bad case of retroflexion of the uterus with fixation and prolapsus of the ovaries under his care in the hospital. He asked me to see the case in consultation with him, which I did.

The overshadowing feature of the case was epileptiform hysteria. I recommended columning of the vagina with dry cotton according to my method, which, at the request of Dr. P., I undertook myself.

After a couple of months of persevering effort, during which time the patient had only one of her old attacks of hysterical convulsions, decided progress was made toward the replacement of the affected organs, and the patient felt she was greatly benefitted. At this stage of the treatment I made a trial of my new vaginal support intended to take the place of the column and to brace the uterus in its partially elevated position by acting on the cervix uteri, the short arm of the lever, and thus to avoid pressure upon the implicated ovaries by the crowding upwards of the posterior *cul de sac*, as with the Hodge's pessary; but the instrument was in a crude state of evolution at that date and failed to meet the ends proposed. It was, therefore, laid aside for the time. In order to adopt some form of support to the ovaries, under these circumstances, I recommended my old and ready expedient, the intra-vaginal sponge cylinder in oil-silk, and I instructed the patient how to use it. During the treatment of this case, quite a number of my medical friends, of this city, visited the hospital to witness my mode of columning the vagina with dry cotton. Among them, Drs. Isaac E. Taylor, Meredith Clymer, Jean F. Chanorin, Monteferro J. Moses and others. Sometime afterwards the patient called to see me at my office, and an examination showed the uterus in about the same stage of elevation in which I had left it, but she was not relieved of her hysterical attacks and was much depressed as to her prospects of recovery. She afterward, I learned, consulted a distinguished gynæcologist of this city, fond of using the knife, and he performed the operation successfully, of extirpating both ovaries. The precise condition of the patient at the present time I cannot state from my own knowledge, but I learn from Dr. Paine that her health has been much improved by this operation.

I will now cite a most interesting case of anteflexion of the uterus as an illustration of the value of my new method for this form of displacement, and to point out its precise mode of application in the recumbent position.

Mrs. P., of this city, aged 30, married ten years, sterile, general health much depreciated by a long train of nervous complications, which had existed almost from the time of her marriage, consulted me November 9th, 1877. She had been under the care of eminent physicians, who had tried a variety of pessaries, none of which, however, she could wear without the greatest discomfort. On examination I found a deep anteflexion of the uterus with slight endometritis and an irritating discharge which had resulted in vaginitis and vaginismus. Having before made applications of my method of dry cotton in similar cases I was led to try it here. The plan pursued was this: The patient resting in the recumbent posture upon a table, bed, couch or reclining chair, my second size speculum is introduced into the vagina and the blades expanded by turning the thumb-screw to the required extent. The detached blade is then slid in upon the perineo-rectal wall and after depression of the latter with it, adjustment is effected upon the projecting arches of the heel of the blades as indicated by Fig. 1. At this stage of procedure the cervix uteri will usually be seen thrust back toward the hollow of the sacrum and the body of the organ presenting forwards and downwards at an angle of about 45° to the axis of the vagina. Next, the spatula double hook, held in the left hand, is planted in the anterior lip of the cervix uteri, the spatula part holding up, at the same time, the urethral portion of the vagina. With this control the whole speculum is now pressed gently backwards with the angular end of the perineo-rectal depressor held between the thumb and index finger of the right hand, at the same time the cervix uteri is drawn downwards and upwards with the hook. Thus the cervix is lifted from its displaced position and brought partially, if not completely within the field of the instrument, when the hook is removed and it settles down securely upon the inner face of the depression. Now the anterior columning begins. The dry cotton pads being prepared as before described, one is seized with my uterine forceps and placed firmly against the center of the vesico-vaginal septum, carrying the latter before it upwards and backwards to the highest point of elevation. While thus pressed up it is caught by my narrow, curved, smooth spatula, held in the left hand, which allows the forceps to be removed, and so ball after ball of cotton is deposited the entire width of the anterior wall of the vagina and held in position. In this manner a firm column is made obliquely downwards and backwards to a point between the body of the blades of the speculum. Here the firmness of the column is increased on account of the interposition of the flaring blades and the protection of the lateral walls of the vagina against undue pressure. This point having been passed, the column is continued on to the surface of the rectal depressor just within the contractile range of the perineum.

The column now being still pressed upwards and backwards with the narrow-bladed spatula, held in the left hand, the perineo-rectal depressor is disengaged and withdrawn with the right hand. Next, by reversal of the screw and the inclining backwards of the collapsed blades, the column becomes disengaged. The speculum is removed and afterwards the spatula. With the removal of the speculum the lower third of the perineo-rectal surface mounts upwards to the support of the newly constructed column. Acting in this way the pressure upon the vesico-vaginal septum through the column is continued upwards and backwards against the body of the uterus, the long arm of the lever. Gradually the anterior wall of the vagina stretches, and with it the utero-vesical ligaments yield. The column is allowed to remain thirty-six hours, when the patient removes it herself with the looped threads left for the purpose, and takes her usual warm vaginal douche. Rest and vaginal douches twice daily are allowed for thirty-six hours, when the column is again introduced. No assistant is required for the procedure.

Here it is again seen that the flaring blades of the speculum fulfill most important ends, as pointed out in connection with the plan previously described for retroversion and retroflexion of the uterus which it is unnecessary to repeat.

Suffice it to say that my columning against the vesico-vaginal septum, in the way described, was kept up at intervals of two or three days for five weeks, with the result of placing the uterus in a good position and restoring the normal contour of the vagina.

Having made important improvements in my vaginal support, since it was used in St. Elizabeth's Hospital five or six months before, I concluded to try it in this case. This I did December 14, 1877, and the result was most satisfactory. The patient wore it with the greatest comfort, and soon the uterus was made to stand alone in its normal position. About three weeks afterward, when her menstrual period was just over, and she was still wearing the vaginal support during the day only, pregnancy occurred. The support was worn a month longer, or until after the time for the next period, when it was thought to be unnecessary. The pregnancy progressed satisfactorily and terminated favorably to mother and child. It is now five years since my treatment was made, and the patient remains perfectly well.

Here then was a successful application of the principle of anterior columning of the vagina with dry cotton for anteflexion of the uterus according to my method, and based upon the idea of pressure which really astonished me. It was upon the principle of the parallelogram of forces that I constructed this *vaginal support*, and it is, I conceive, only by a just appreciation of this law in mechanics that my form of pessary or support, intended for the vagina, can be made to subserve its highest aims. It was devised with the idea of supplementing the use of the columns in all forms of vaginal, uterine and ovarian displacements, resulting from whatever cause.

In February, 1878, when I was appointed surgeon to the Woman's Hospital of the State of New York, I found a broader field for observation and particular study of the class of diseases under consideration than I had before enjoyed. I now introduced into this institution my peculiar methods of treatment regarding not only the graver, perforating lesions of the organs concerned in paturition, but the milder ones resulting from changes in former relationship and function. Prominent among these methods were dilatation of the vagina for cicatricial and contracting bands as complications of vesico-vaginal fistule (kolpostenosis) with hard rubber and sponge dilators, and the columning of the vagina with dry cotton, together with my speculum and supporting and confining apparatus for the knee-chest position. With regard to the columning of the vagina, especially, the old nurse who had been connected with the institution almost since its foundation was quite amazed, not only as to the quantity of cotton I used, but at the facility with which I disposed of it without her valuable assistance as a speculum holder, which, until then, she had believed to be absolutely indispensable for all forms of vaginal and uterine treatment.

For the innovation of using dry cotton she claimed the right to dub my dry cotton balls with looped threads, "snow-balls" in contradistinction to the old form in use there, "the butterfly"—a single pad of cotton saturated with glycerine and likewise secured with a loop of thread for removal, intended for medicated applications, alone, to the cervix uteri. Not only this, but the suddenly increased expenditure for cotton was such as to attract the attention of the Lady Managers and to excite comment.

As to what I have accomplished in the Woman's Hospital by my peculiar methods of practice, designated these sometimes, "the new element" it is not necessary for my present purpose to indicate. It is a matter of record and is well known to a large class of physicians scattered over our country. So much, then, for my understanding the principle of using dry cotton in the treatment of diseases of the uterus and ovaries at this date.

[TO BE CONTINUED.]

## Figures and Tables

**Fig. 1. f1:**
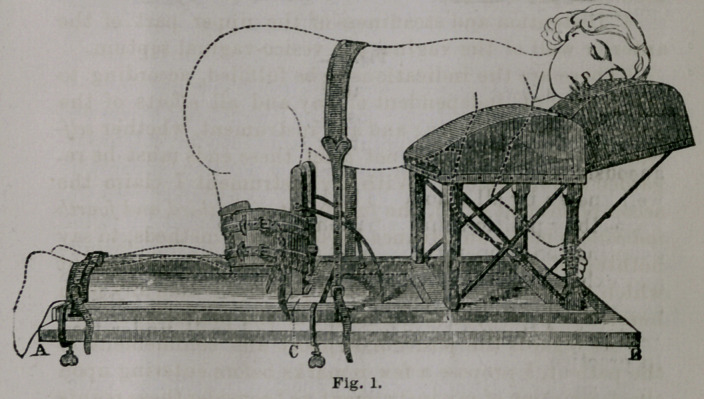


**Fig. 2. f2:**
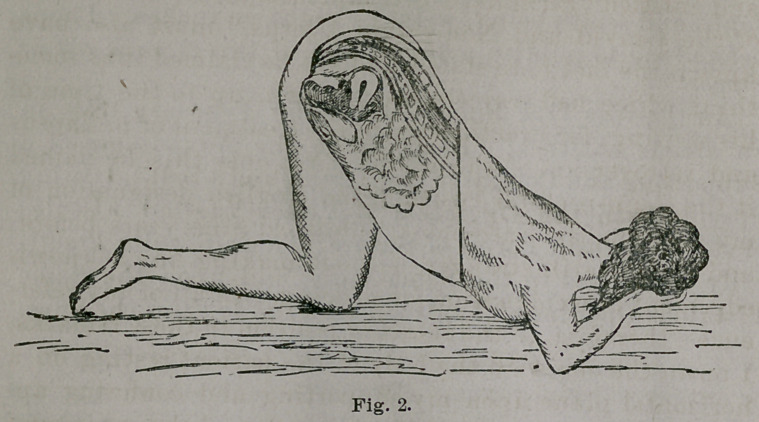


**Fig. 3. f3:**
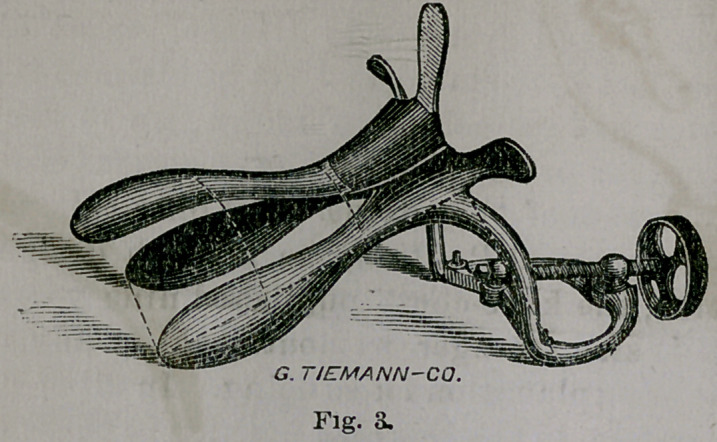


**Fig. 4. f4:**